# Selective enzymatic debridement and modified Meek technique in the treatment of extensive burns: Preliminary observations

**DOI:** 10.1002/hsr2.1829

**Published:** 2024-07-21

**Authors:** Jasminka Minic, Enrico Vigato, Yaron Shoham, Umberto Lavagnolo, Maurizio Governa

**Affiliations:** ^1^ Burn Center University Hospital of Verona Verona Italy; ^2^ Burn Unit, Soroka University Medical Center, Faculty of Health Sciences Ben‐Gurion University of the Negev Beer Sheba Israel

**Keywords:** burn units, cicatrix, humans, skin transplantation, surgical mesh

## Abstract

**Background:**

Selective bromelain‐based enzymatic debridement (BED) has emerged as a valid alternative for the treatment of extensive burns, with Total Body Surface Area (TBSA) > 20%. Autologous skin grafting represents the procedure of choice but the scarcity of donor sites remains the main reconstructive challenge. The modified Meek micro‐grafting technique may represent a valid strategy to optimize the final outcome.

**Methods:**

A single‐cohort retrospective analysis was performed, involving nine burn patients (TBSA > 20%) who underwent both BED and subsequently modified Meek technique. Demographic and clinical data (mechanism of injury, surgical treatment, complications, necessity of re‐grafting, further surgery and esthetic outcome) were collected.

**Results:**

All patients had large burns of mixed and deep dermal thickness (first, second, and third degree). All burns were enzymatically debrided postadmission and covered by the modified Meek technique. Local infection due to poor general conditions was the main complication for all patients. All but two patients survived. The selectiveness of the enzymatic debridement and dermal preservation seemed to improve the quality of scars resulting from micro‐grafting. Evaluations performed at 12 ± 2 months postburn showed superior scar quality compared to areas treated with traditional (sheet/mesh) grafts.

**Conclusion:**

Combined BED and Meek techniques may provide an effective synergic combination for the treatment of extensive burns.

## Key messages

- Combined bromelain‐based enzymatic debridement and Meek techniques may provide an effective synergic combination for the treatment of extensive burns, implying superior scar quality.
- Nine burn patients were evaluated, demographic, epidemiological and clinical data were collected.
- The selectiveness of the enzymatic debridement and dermal preservation seemed to improve the quality of scars resulting from micro‐grafting.

## 1 INTRODUCTION

The treatment of patients suffering from cutaneous burns has undergone a significant innovation in recent years. The introduction of bromelain‐based enzymatic debridement (BED) represents a valid alternative to traditional surgical debridement (escharectomy) and for the release/prevention of elevated burn induced compartment pressure in patients suffering from deep or mixed pattern burns (first, second and third degree of tissue involvement).^1^ The recent European and national consensus guidelines for the management of this emergency therapy,^2^, ^3^ propagated BED debridement to an increasing number of hospital‐based burn centers. Several studies have addressed the benefits of the selective enzymatic debridment.^4^, ^5^ The main advantages include: reduced debridement related blood loss, reduced number of wounds requiring surgical excisions; reduced number and area of autologous skin grafts; reduced wound infections and reduced hospitalization times.^1^, ^6^ On the contrary, BED is contraindicated in patients with a known hypersensitivity to anacaulase‐bcdb, bromelain and pineapples.^1^

Though BED application is limited to 15% total body surface area (TBSA),^3^ it is common practice in experienced burn centers to use BED to treat larger % TBSA burns (off label use). The challenge in these patients is to cover the enzymatically debrided exposed deep dermal and full thickness defects.

The classic approach is to graft the debrided areas with split‐thickness autografts harvested from the patient's noninjured areas. In patients with extensive burns the limited donor skin surface necessitates expansion of the autograft to ensure coverage of the large exposed sites.^7^ In patients with very limited donor site the meshing of small autografts is challenging, often resulting in inferior quality grafts, reducing graft take, increasing the risk of local superinfection and delaying re‐epithelialization.^8^ The Meek technique is a method of processing skin graft tissue based on the use of a partial‐thickness skin expansion device, called a micrograft. This technique allows for an increased expansion rate and easier handling of small and larger autografts.^9^ Kreis et al. modified the technique offering an easier manipulation and a greater consistency of the result, through the use of special prefolded gauzes.^10^ Although this autograft technique has been known for many years, we are not aware of studies or reports in the literature describing the treatment of a burn patient undergoing BED combined with the Kreis modified Meek technique (MMT) for covering burn areas.

We have been treating extensive burns with this approach since 2018 in our Operating Unit Complex ‐ Burn Center of the Integrated University Hospital of Verona, Italy. The aim of this preliminary retrospective, single cohort, observational study is to assess the potential advantages of combining BED and engraftment by MMT in the treatment of patients affected by extensive burns, defined as involving >20%TBSA.

## 2 METHODS

This is a retrospective analysis of a single cohort of medical records and posthospitalization outpatient follow‐up visits of nine patients with severe burns involving >20%TBSA over the years 2018–2021. These patients underwent one‐stage enzymatic debridement with NexoBrid® (MediWound Ltd.) and were subsequently autografted according to the MMT. Initially, allergy compatibility on an anamnestic basis was assessed before the application of BED. The application procedure of the enzymatic product was carried out in the operating room, under general anesthesia and for a standard time of 4 h. Subsequently, the excarolytic product was removed completely as per protocol, allowing the burned areas to be exposed for treatment with skin grafts. The goal of graft application was complete coverage of all affected surfaces except for decubitus areas where the grafts would induce dehiscence. MMT skin grafts were applied in the central regions of the burns, while traditional non‐Meek skin grafts were applied in the peripheral areas near the healthy skin.

After coverage, the lesions were dressed with paraffin‐coated gauze and sterile bandage gauze. Bandage dressings were renewed daily and graft coverage was performed approximately 7–15 days after debridement. The following data of each patient were retrospectively standardized and assessed: demographics; burn anatomical sites and surface %TBSA); local pathogens superinfection; mortality; date, number and type of surgical procedures; the Meek ratio that defines the graft augmentation (obtainable by processing skin tissue according to Meek technique) versus the donor site surface harvested; the percentage of local graft failure; the percentage of skin surface covered by the MMT; qualitative and quantitative esthetic outcome.

The assessment of long term scarring outcomes was done a year postinjury by using the *Vancouver Scar Scale* (VSS) and the *Patient Scar Assessment Scale* (PSAS) from the multitude of evaluation scales for quantifying scar quality and its impact on cognitive and psychological health.^11^, ^12^, ^13^ The data obtained were re‐elaborated and interpreted considering the mean value and the corresponding standard deviation for each scale, comparing the areas treated with MMT and those treated with traditional grafts. The results were statistically evaluated by applying the two‐sided student's T‐test and statistical significance was defined with *p*‐value < 0.05 (calculation program used: Prism 8 – GraphPad®). Finally, a qualitatively comparison with other studies in the literature was performed.

Treatment with NexoBrid® falls within the hospital protocols where the study was conducted, being approved by the Hospital Pharmacy Service and being available in the formulary. The grafting techniques fall within the Best Practice procedures of the Plastic Surgery Unit ‐ Burn Center of the University Hospital of Verona. In particular, the Meek technique is already considered a procedure comparable to traditional skin grafting, as no comparable treatments are available. The Local Ethics Committee approved the study as the study procedures were already individually validated for the described use (n. 56131 – 30/11/16).

## 3 RESULTS

Nine patients were enrolled in the study, all suffering from large surface deep flame burns, mixed dermal and/or full thickness in depth (III degree^14^), with some burns involving the subcutaneous tissues. The grading of burn involvement was reported in Table 2, with reference to the different anatomical burn regions. All patients were admitted to the emergency room of our hospital and promptly evaluated by a multidisciplinary burn team (emergency medicine specialist, anesthetist and plastic surgeon). Seven patients underwent BED application in the first 24 h after injury, and two underwent treatment within 24–48 h after injury (Table 1). The different timing of the treatment can be attributed to anesthesiological issues that did not allow the patient to undergo the operating session. The age of the patients varied over several age‐groups, with an average age of 46.6 ± 22.3 years. Five patients were male and 4 were female. The mean surface area burned was 44 ± 15%TBSA. The burned anatomical areas are summarized in Table 2. No patients developed compartment syndrome before, during and after the performed excarolytic treatment. Two patients expired during late hospitalization, approximately 4 months postinjury, due to cardio‐ventilatory decompensation caused by complications not directly related to burns. Positive microbiological swabs of the burns were found in all patients except one, with different pathogens also detected in the same subject. In particular, *Acinetobacter baumanii* was the most commonly found bacteria, found in 6/9 of the patients. Areas of dehiscence were treated surgically with debridment in combination with intravenous antibiotic therapy. Notably, all patients with findings of local overinjection were monitored by infectious disease physicians.

**Table 1:** Summary of the collected database. The study involved nine patients treated at our Burn Center from 2018 to 2022.

	Age	Sex	TBSA	Mortality	Infection	N° interventions	Meek ratio	% Meek areas	Meek + bed absence of relief texture	% Post Meek dehiscence	N° Re‐interventions in meek‐treated areas	N° Re‐interventions in non meek‐treated areas
**1**	66	F	30%	✗	*S. epidermidis*; *Pseudomonas putida*; *S. hemolyticus*; *Morganella morganii*; *C. albicans*; *S. coagulasi* neg; *Acinetobacter baumanii*	3	1:6	80%	✓	7%	1	0
**2**	24	M	65%	✗	*Acinetobacter baumanii*, *Klebsiella pneumoniae*; *Pseudomonas aeruginosa*	5	1:6 + 1:4	75%	✓	16%	2	1
**3**	56	M	27%	✗	*S. epidermidis*	3	1:6	80%	✓	5%	1	0
**4**	27	F	60%	✗	*Acinetobacter baumanii*	5	1:6	70%	✓	6%	0	2
**5**	26	F	35%	✗	*Acinetobacter baumanii*	7	1:6	75%	✓	10%	1	0
**6**	86	M	30%	✓	—	3	1:6	85%	✓	18%	1	0
**7**	32	M	50%	✗	*E. cloacae*; *Acinetobacter baumanii*; *S. epidermidis*; *S. haemoliticus*	3	1:6	75%	✓	8%	1	0
**8**	65	F	35%	✗	*E. coli*; *S. aureus*; *Acinetobacter baumanii*; *Elisabethkingae meningoseptica*	4	1:6	90%	✓	10%	1	0
**9**	37	M	60%	✓	*Pseudomonas aeruginosa*; *Staphylococcus haemolyticus*; *Enterococcus faecium*; *Candida parapsilosis*	4	1:6 + 1:4	85%	—	15%	1	0

Abbreviations: BED, bromelain‐based enzymatic debridement; TBSA, total body surface area.

**Table 2:** Anatomical location, gradings (I grade, II grade, III grade) of burns and the total body surface area (TBSA) reported for each patient.

	1	2	3	4	5	6	7	8	9
Face/scalp			X (I)	X (II)			X (I)		X (I)
Neck	X (II)			X (II)					
Chest	X (III)	X (II)	X(II)	X (III)				X (II)	X (III)
Upper limbs	X (III)	X (III)		X (III)		X (III)	X (III)		X (III)
Back	X (III)						X (II)	X (III)	X(II)
Abdomen		X (II)	X (III)		X (III)	X (II)		X (III)	X (III)
Perineum			X (III)		X(III)			X (II)	
Lower limbs		X (III)	X (III)	X (III)	X (III)	X(II)	X (III)	X (III)	X (III)
TBSA	30%	65%	27%	60%	35%	30%	50%	35%	60%

All patients underwent multiple surgical procedures, undergoing multiple skin grafts, with at least one traditional (mesh or sheet) autografting surgery performed after MMT treatment. The mean number of interventions performed for each patient was 4.1 ± 1.4. The timing of the MMT after BED treatment was after 1 week in two patients, after 3 weeks in one patient, after 4 weeks in two patients, after 5 weeks in two patients, and after 7 weeks in one patient. In a single case, two MMT treatments were performed 2 weeks apart. A Meek ratio of 1: 6 was used in all patients. In three patients, part of the donor sites were also treated with a Meek ratio of 1:4 and in a single patient with a Meek ratio of 1:9. The donor site was chosen considering the injured anatomical areas, with preference for the lower extremities (6/9) over the upper extremities (2/9) or the abdomen (1/9). More than one donor sites were used in all but one patient (abdomen donor site). The size of the MMT grafted areas was on average 78.5% ± 5.2% of the burned skin. Graft failure was on average 11% ± 4.7% of the total grafted area in all patients that needed additional closure by sheet/mesh autografts (an additional 1 ± 0.5 procedures). In particular, the reoperations were conducted with a traditional (non‐Meek) grafting technique.

**Figure 1:**
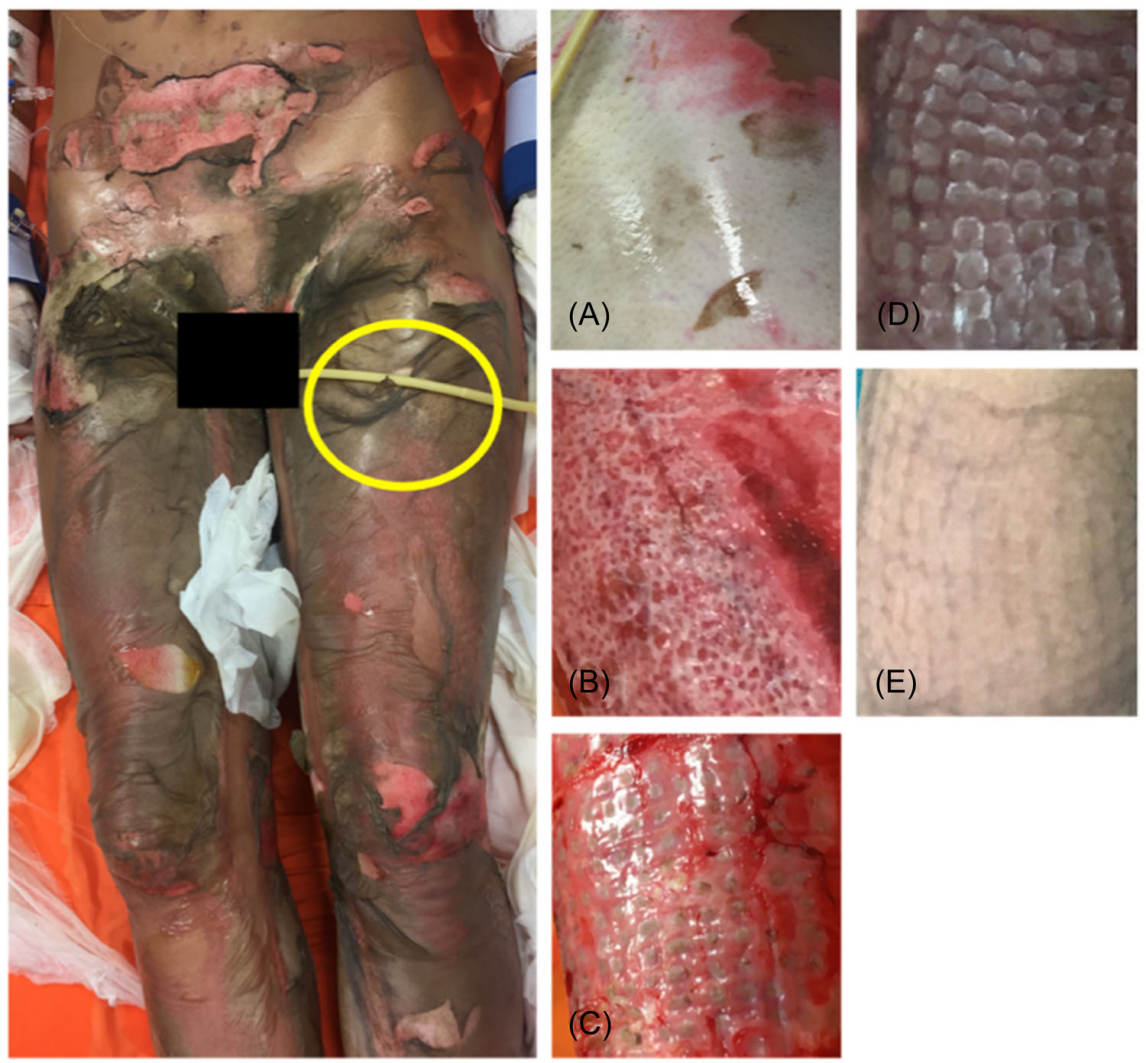
26‐year‐old female patient, suffering from flame burns involving 35% total body surface area (TBSA). (A) Detail of the burned thigh. (B) Same area after bromelain‐based enzymatic debridement treatment (BED). (C) Skin graft treated with modified‐Meek technic (MMT) 2 weeks after surgery. (D) Revaluation 2 months after grafting. (E) 1‐year revaluation. Note a scar with no quadrangular texture detected. The patient provided consent for the photos to be published. Courtesy of the Burn Center ‐ Integrated University Hospital of Verona, Italy.

**Figure 2:**
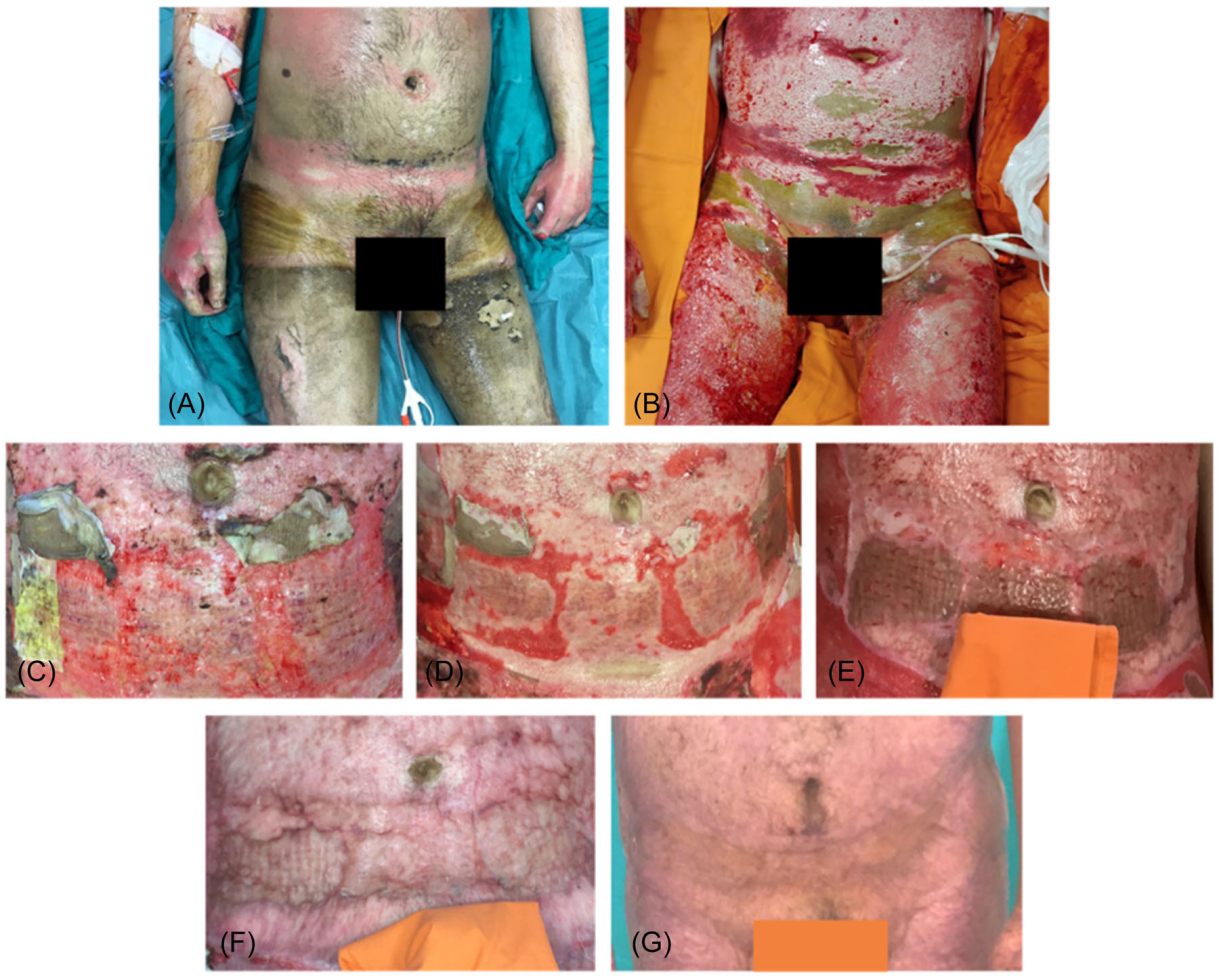
(A) 56‐year‐old male patient, suffering from flame burns involving 27% total body surface area (TBSA). (B) After treatment with Bromelain‐based enzymatic debridement (BED). (C) Same area after skin graft treated with modified‐Meek technic (MMT). The homografts applied to the bloody areas are noticeable. (D) After 2 weeks. (E) 1 month after grafting. (F) Three month follow up. (G) One‐year follow up. Note the scar of the treated region is devoid of detected quadrangular texture. The patient provided consent for the photos to be published. Courtesy of the Burn Center ‐ Integrated University Hospital of Verona, Italy.

**Figure 3:**
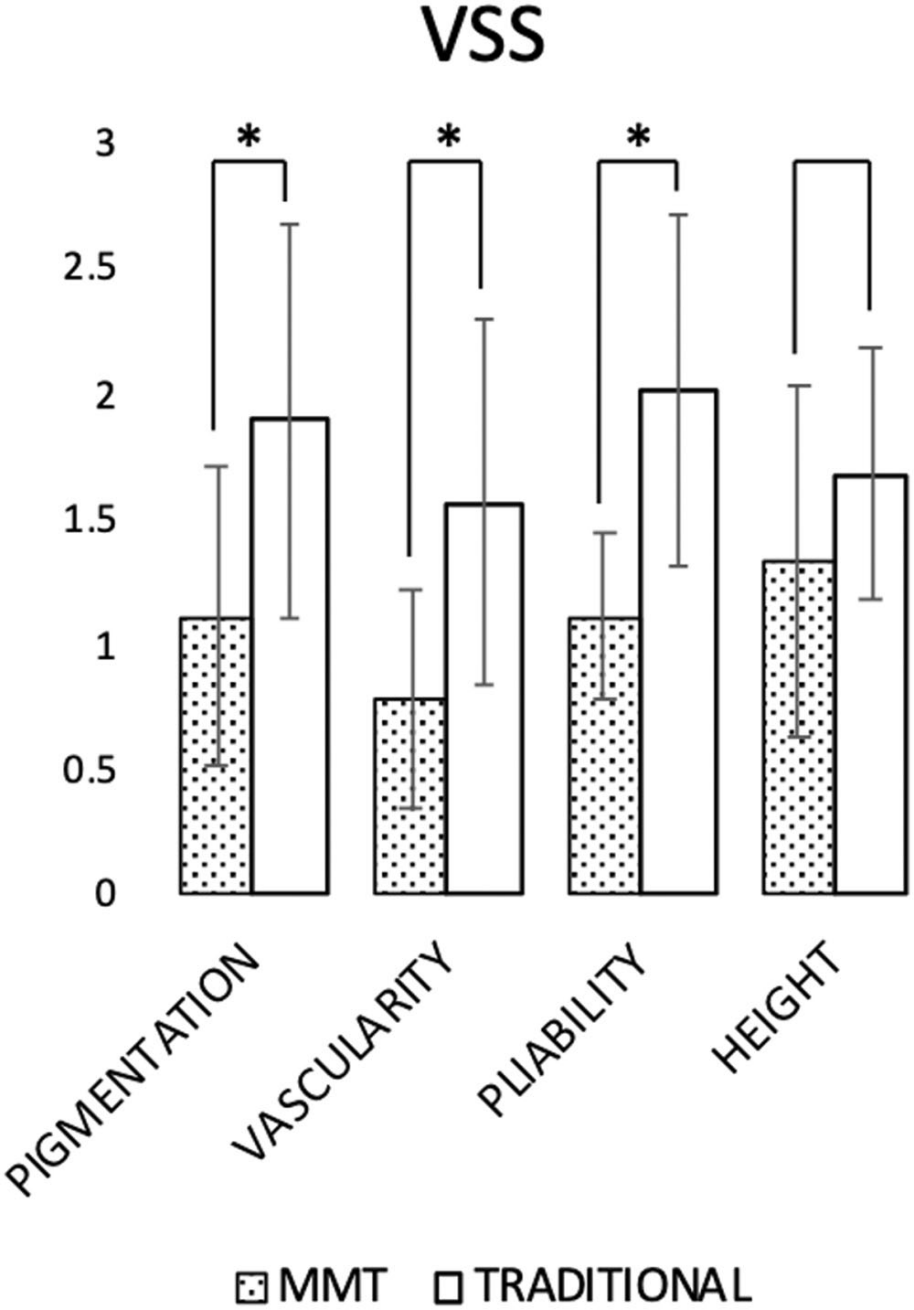
One year follow‐up Vancouver Scar Scale (VSS) mean ± SD scores. (* = *p* < 0.05).

Postoperative monitoring and serial outpatient visits demonstrated good healing of the burned areas treated with MMT following BED (Figures 1 and 2). Evaluations performed at 12 ± 2 months postburn showed superior scar quality compared to the areas treated with BED + traditional grafts. Skin regions not affected by the burn were considered as the standard of comparison. The scars of the areas engrafted by MMT did not have the expected raised quadrangular texture, were elastic and moved freely over the deep planes. The scarring as measured by VSS result was satisfactory (Figure 3) regarding skin pigmentation (1.11 ± 0.6 for MMT vs. 1.89 ± 0.78 for traditional grafts), vascularization (0.78 ± 0.44 for MMT vs. 1.56 ± 0.73 for traditional grafts), pliability (1.11 ± 0.33 for MMT vs. 2.00 ± 0.71 for traditional grafts) and scar thickness (1.33 ± 0.7 for MMT vs. 1.67 ± 0.50 for traditional grafts).

**Figure 4:**
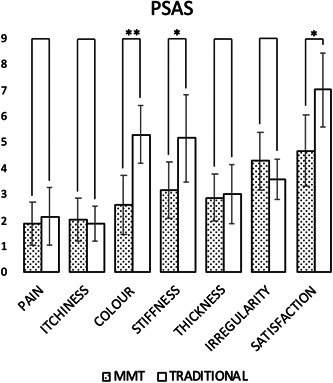
One year follow‐up Patient Scar Assessment Scale (PSAS) mean ± SD scores. (*  = *p* < 0.05, ** = *p* < 0.005).

The patients reported greater satisfaction of the final scars outcomes of areas treated with the BED + MMT combination as measured by PSAS compared to those treated with the BED + traditional graft combination. The data collected were the following: local pain (1.85 ± 0.82 MMT vs. 2.14 ± 1.11 traditional grafts), itch (2.00 ± 0.82 MMT vs. 1.86 ± 0.69 traditional grafts), color (2.57 ± 1.13 MMT vs. 5.29 ± 1.11 traditional grafts), stiffness (3.14 ± 1.07 MMT vs. 5.14 ± 1.68 traditional grafts), thickness (2.86 ± 0.90 MMT vs 3.00 ± 1.15 traditional grafts), irregularity (4.29 ± 1.11 MMT vs. 3.57 ± 0.79 traditional grafts) with an average good satisfaction (4.67 ± 1.37 MMT vs. 7.00 ± 1.41 traditional grafts) (Figure 4).

## 4 DISCUSSION

Treatment of patients with extensive burns remains a clinical and surgical challenge.^15^, ^16^ In the acute phase maintaining homeostasis (fluid balance, effective ventilation and perfusion) is critical for survival. However, local care of the injured skin is not less important for the following course. Ensuring optimal damage control, as early as possible debridement of the nonviable tissues and adequate skin coverage and wound closure of the injured areas will define the healing course with its potential lethal complications (infection, sepsis) and final outcome of quality of life (cosmesis and function).^17^ Effective, early and selective BED (escharectomy) has become a fundamental tool for the first step of burn wound care. Early removal of the dead tissues prevents eschar related complications as well as resolving or preventing burn induced compartment syndrome with its additional risks. The debrided bed should be covered to protect it from additional risks (desiccation and infection) preferably with a permanent cover‐graft that will close the raw wounds.^18^ Autografting is the best solution for permanent closure of the debrided wounds but it depends on the availability of healthy donor site skin. The larger the burns, the less donor sites and autografts are available, necessitating using techniques to increase the size of the grafts (meshing and Meek micrografts). These techniques rely on the “take” of the meshed or micrografts and epithelialization of the spaces within the mesh or between the micrografts. In surgically debrided burns the bed is very often a full thickness defect leading to necessity for extensive grafting. Performing BED, wound beds many areas have dermal remnants that allow for some spontaneous epithelialization and better healing. In addition, the quality and size of the harvested grafts often challenge its meshing leading to poor quality grafts and irregular scarring at the edges and within the grafts, ending with disfiguring, raised and rigid scars.^19^ A BED offers a potential for early closure that in extensive burns is limited by insufficient donor sites and the resulting limited autografts. The use of MMT may be a practical answer to this limitation as demonstrated in this study.

The cohort in this study contains patients of different age groups and gender with extensive burns in different anatomical sites. In all these cases, BED was applied within 48 h from injury, according the instructions for use.^2^, ^3^ The coverage with MMT was done in different timings depending on the wound bed condition. Some publications suggest permanent cover (when needed) within a few days postdebridement and others suggest delaying permanent autograft closure for more than 2 weeks in the absence of satisfactory re‐epithelialization.^2^ Early enzymatic debridement reduces the risk of contamination and superinfection of burn eschar, allowing delayed grafting with reduced risk of infection/inflammation that may slow or complicate it.^20^ The care of extensive burns usually involves multiple interventions from serial debridement to several autografting procedures, which is limited by the scarce donor site area. An autograft method that provides a high take rate of large grafted areas and good quality epithelialization of the spaces between grafts and mini and micro‐grafts (as MMT) represents an important useful tool in burn care with the potential of also reducing healthcare costs.

The expansion of the autograft beyond the size of the available donor site to cover large burn areas represents a significant advantage over the classic non expanded sheet grafting technique. The expansion can be adjusted to needs (quality of final healed wound, quality and quantity of available autograft) by meshing ratios (1/1 up to 1/9) but small and irregular pieces of autograft cannot be meshed in wide ratios. Such small autografts can be easily cut to Meek micrografts to all ratios. In our clinical practice, the most used Meek ratio was 1: 6 (used in 7/9 patients), with 1: 4 used in only two cases allowing us to cover all the debrided burns. The rate of graft failure of the individual micrografts of the MMT grafting was limited and in line with other similar studies in the literature.^7^, ^21^ The skin grafts treated according to the MMT were placed in the central areas of the lesions, while in the peripheral regions adjacent to healthy tissue, traditional non‐Meek skin grafts were applied. Specifically, in the tissues most damaged by the injury, the skin islands of the Meek technique allowed for good graft survival despite the severe compromise of the subcutaneous tissue and vascular structures. Unlike traditional grafts, micrografts are characterized by a reduced nutritional demand from the transplanted tissues, largely avoiding graft dehiscence due to insufficient vascular supply.^22^, ^23^ In the peripheral areas with shallower lesions and partially intact vascularization, the massive nutrient demand from the graft may have been supported, thus preventing dehiscence.

In clinical experience extensive burns such as in this cohort require numerous interventions (surgical debridement, graft harvesting and engraftment) to ensure wound closure and adequate final outcome. Several risks are related to these procedures, mainly general anesthesia risks, including significant blood and heat losses. In our experience BED offered an early and effective eschar removal with little or no need for general anesthesia and practically no procedure related blood and heat losses. Besides the obvious benefit to the patient reducing these procedures also helps reducing healthcare costs.

The final outcome of scarring assessed by VSS and PSAS showed good clinical and functional results, as well as good patient satisfaction. Healthy skin regions unaffected by the burn, located possibly near the burned areas, were considered as the standard of comparison. The scars of the areas grafted with MMT were characterized by good pliability and color. Due to clinical requirements and in view of the extensive burned areas, it was not possible for all patients to harvest from donor areas with clear skin pigment. Despite this aspect, patient satisfaction with this criterion was qualitatively good. In particular for VSS (Figure 3), the data analysis highlighted a statistically significant difference between the areas treated with MMT and those treated with traditional grafting for all items of the scale, except for height. As for the PSAS (Figure 4), a statistically significant difference between the two treatments was found only in reference to color (*p*‐value < 0.005), stiffness and general satisfaction. This could be interpreted as a good result for the MMT associated with the application of BED: there is no increased thickness compared to the traditional graft, however obtaining an improvement in terms of color, vascularity and pliability. Furthermore, the chromatic difference was the most impacting element for the patient, finding greater satisfaction from the areas treated with MMT.

This retrospective, single cohort study is undoubtedly affected by a limited possibility of generalizing the data validity and the difficulty of establishing a cause‐effect relationship, also considering the small sample analyzed (*N* = 9). In fact, a considerable limitation of this evaluation is the extreme variability of the clinical result for the individual patient, in relation to the different burned areas (not necessarily with the same stage) treated with the two techniques. Furthermore, the surgical times were not homogeneous for all patients. In light of this, we believe that the general assessment reported by us can take a qualitative rather than a quantitative indication. Further studies will be needed to improve the uniformity of the analyzed cohort.

One hallmark of Meek micrografts is the raised areas of the square micrografts above the spaces around them due to contracture of scars forming at the edges of the dermal‐epidermal graft (“purse‐string phenomenon”) and depressed epithelialized spaces around it. These results stem from the fact that Meek grafts are usually applied on subcutaneous fat or granulating subcutaneous fat. This wound bed is not easily epithelialized from the micrografts' edges, thus allowing for inflammation, granulation and scarring before complete epithelialization takes place. BED leaves viable dermal and vascular remnants that can serve as a matrix for faster epithelialization from the micrografts' edges, thus reducing the interspaces scarring resulting in a smoother, uniform appearance and a softer, pliable, nonadherent end result. This process of healing with minimal or no inflammation is enhanced by the well documented strong anti‐inflammatory properties of Bromelain.^24^

While this is a very small patient cohort presenting preliminary results with this combined technique, we believe this study demonstrates a potentially beneficial, synergic activity in extensive deep burns of these two standard procedures: early and selective BED and wound closure by MMT.

## 5 CONCLUSION

BED combined with autologous MMT dermal‐epidermal micrografting seems to be a valid treatment option for patients suffering from extensive burns. Additional studies are needed, with larger cohorts and control arms.

## AUTHOR CONTRIBUTIONS

**Jasminka Minic**: Conceptualization; data curation; investigation; methodology. **Enrico Vigato**: Formal analysis; investigation. **Yaron Shoham**: Data curation; formal analysis; methodology; supervision. **Umberto Lavagnolo**: Data curation; formal analysis; writing—original draft; writing—review and editing. **Maurizio Governa**: Supervision; validation.

## CONFLICT OF INTEREST STATEMENT

All authors declare that they have no conflict of interest.

## TRANSPARENCY STATEMENT

The lead author Umberto Lavagnolo affirms that this manuscript is an honest, accurate, and transparent account of the study being reported; that no important aspects of the study have been omitted; and that any discrepancies from the study as planned (and, if relevant, registered) have been explained.

## Data Availability

The data that support the findings of this study are available on request from the corresponding author. The data are not publicly available due to privacy or ethical restrictions.
